# Artificial Cervical Vertebra and Intervertebral Complex Replacement through the Anterior Approach in Animal Model: A Biomechanical and *In Vivo* Evaluation of a Successful Goat Model

**DOI:** 10.1371/journal.pone.0052910

**Published:** 2012-12-27

**Authors:** Jie Qin, Xijing He, Dong Wang, Peng Qi, Lei Guo, Sihua Huang, Xuan Cai, Haopeng Li, Rui Wang

**Affiliations:** The Department of Orthopedics, the Second Hospital of Xi'an Jiaotong University, Xi'an, Shaanxi Province, People's Republic of China; University of Zurich, Switzerland

## Abstract

This was an in vitro and in vivo study to develop a novel artificial cervical vertebra and intervertebral complex (ACVC) joint in a goat model to provide a new method for treating degenerative disc disease in the cervical spine. The objectives of this study were to test the safety, validity, and effectiveness of ACVC by goat model and to provide preclinical data for a clinical trial in humans in future. We designed the ACVC based on the radiological and anatomical data on goat and human cervical spines, established an animal model by implanting the ACVC into goat cervical spines in vitro prior to in vivo implantation through the anterior approach, and evaluated clinical, radiological, biomechanical parameters after implantation. The X-ray radiological data revealed similarities between goat and human intervertebral angles at the levels of C2-3, C3-4, and C4-5, and between goat and human lordosis angles at the levels of C3-4 and C4-5. In the in vivo implantation, the goats successfully endured the entire experimental procedure and recovered well after the surgery. The radiological results showed that there was no dislocation of the ACVC and that the ACVC successfully restored the intervertebral disc height after the surgery. The biomechanical data showed that there was no significant difference in range of motion (ROM) or neural zone (NZ) between the control group and the ACVC group in flexion-extension and lateral bending before or after the fatigue test. The ROM and NZ of the ACVC group were greater than those of the control group for rotation. In conclusion, the goat provides an excellent animal model for the biomechanical study of the cervical spine. The ACVC is able to provide instant stability after surgery and to preserve normal motion in the cervical spine.

## Introduction

Degenerative disc disease (DDD) includes herniated discs and spondylosis in the cervical spine that causes axial neck and/or radicular arm pain and neurological symptoms. DDD is one of the most common spinal disorders and presents a major disease burden worldwide. Great efforts have been made to halt or reverse the disease process via surgical and nonsurgical treatments.

Anterior cervical discectomy and fusion (ACDF) has been considered the standard treatment for the surgical treatment of patients with cervical DDD that is refractory to conservative treatment. ACDF is indicated either when at least 6 weeks of conservative treatment is unsuccessful, when severe neurological symptoms exist, or when patients become unable to perform activities of daily living.

Postoperative dysphagia, hematoma, and symptomatic recurrent laryngeal nerve palsy are the most common complications related to the anterior cervical surgical approach, while esophageal perforation is the most serious complication [1]. The potential morbidities related to ACDF include the possibility of decreased total cervical range of motion, pseudarthrosis, graft donor morbidities (pain, infection, and hematoma), and adjacent segmental degeneration (adjacent disc degeneration, adjacent disc herniation, instability, spinal stenosis, spondylosis, and facet joint arthritis).

Some biomechanical results have shown that the range of motion, intradiscal pressures, and facet joint stresses at adjacent segments may increase due to the loss of motion at the fused segment after fusion [2], [3]. This motion loss is thought to result in accelerated degeneration and mechanical instability in adjacent levels [2], [4]. Radiological changes, such as spondylosis and instability at the adjacent levels, which are not always consistent with the clinical symptoms have been reported by several authors. Several studies has suggested an increased rate of adjacent segmental degeneration after ACDF that may need further surgery [5], [6], [7]. Hilibrand et al [5] have reported that adjacent level degeneration with new radiculopathy occurs in 2% to 3% of patients per year after ACDF and approximately 25% of these patients would suffer from further degenerative disease at adjacent levels within 10 years after ACDF. Although controversy surrounds the issue of whether the postfusion degeneration of adjacent segments is attributable to the natural progression of the disease or to postsurgical biomechanical changes, motion-preserving technology, which allows the preservation of the mobility of the implanted level, will lead to fewer biomechanical changes. The motion-preserving technology could reduce the hyper mobility, intradiscal pressure, and facet joint pressure sustained by the adjacent segments, and therefore, may avoid, or at lease slow down the adjacent segmental degeneration [8].

Anterior cervical disc replacement (ACDR) is an alternative surgical procedure that may replace cervical fusion in selected patients suffering from cervical DDD. The goal of disc arthroplasty is to restore the intervertebral disc and prevent the recurrence of nerve root compression. The theoretical advantages of ACDR are to restore normal motion and to share load with the disc and the facets, which could obviate or reduce the probability of adjacent segmental degeneration, which is the main potential morbidity associated with ACDF. Although a variety of artificial cervical disc devices (e.g., ProDisc-C, PRESTIGE, and Bryan cervical disc prosthesis) are currently being evaluated in FDA-controlled Investigational Device Exemption clinical trials, no artificial disc devices have been approved by the FDA. The indications for ACDR are similar to those for ACDF, but ACDR has strict criteria pertaining to the intervertebral disc and vertebral body. ACDF can not be performed when a patient has more than two vertebral levels that require treatment, cervical instability, cervical fusion adjacent to the level to be treated, prior surgery at the level to be treated, a posttraumatic vertebral body deficiency or deformity, or severe spondylosis [9].

To address this issue, we designed a novel artificial cervical joint, called artificial cervical vertebra and intervertebral complex (ACVC), which can establish spinal stability while maintaining the motion of the surgery segment. In the present study, goats were used to establish an animal model of ACVC implantation. Anatomical and radiological data were collected from goat cervical spines. Based on the goat cervical vertebrae data, we created an ACVC for goat and implanted the ACVCs into goat spines in vitro and in vivo. We then evaluated the safety, validity, and effectiveness of the ACVC through clinical evaluation, radiological evaluation, and biomechanical tests.

## Materials and Methods

### Study design

In this study, radiological and anatomical measurements were performed in 30 fresh adult Chinese white goat cervical spines without skeletal abnormalities, and radiological measurements were performed in 50 normal human cervical spines to acquire the dimensions of the cervical spine in both species. The study was approved by the Ethics Committee of the Secondary Hospital of Xi'an Jiaotong University according to the principles expressed in the Declaration of Helsinki (Approval Number: 2010-16) and the individual in this manuscript has provided written informed consent though the radiological data of human cervical spine was anonymous. Using the radiological and anatomical data, we designed a novel cervical joint, namely an ACVC that both immediately stabilizes cervical spines with anterior cervical discectomy and subtotal corpectomy and allows specific movement of the cervical segments.

ACVCs were implanted into 16 fresh adult Chinese white goat cervical spines in vitro to compare stability and movement in the intact state (control group) with stability and movement after ACVC implantation (ACVC group) before and after the fatigue test. The purpose of this in vitro implantation was to preliminarily evaluate the biomechanical properties of this novel cervical joint in goat spines.

After the in vitro implantation, we established an in vivo goat model with anterior cervical discectomy, subtotal corpectomy, and ACVC implantation. Sixteen Chinese white goats were employed and kept for 12 weeks, and clinical observation, radiological studies (i.e., X-ray films, computed tomography scans and magnetic resonance imaging) and biomechanical evaluations were performed. The purpose of this in vivo implantation was to establish an animal model for the implantation of ACVC and to test the safety, validity, and biocompatibility of ACVC after in vivo implantation.

All of the animal experiments were performed in compliance with the regulations of the Chinese legislation for animal research, and the Animal Care and Use Committee of the Second Hospital of Xi'an Jiaotong University approved the study protocol (Approval Number: 2010-08). All surgery was performed under anesthesia, and all efforts were made to minimize suffering.

### Radiological and anatomical measurements of goat cervical spines

**Figure 1:**
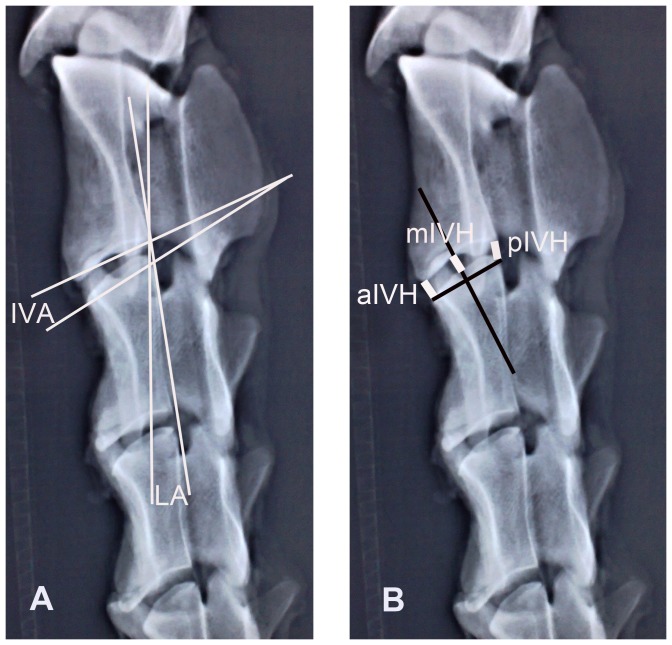
Radiological measurements of goat cervical spines. A, Intervertebral angle (IVA) and lordosis angle (LA); B, anterior, middle, and posterior interverterbral height (aIVH, mIVH, and pIVH).

Thirty fresh goats (age, 24±6 months; average weight, 54.6±3.8 kg; equal numbers of females and males) were used in this study. Their cervical spines (C1–C7) without bony abnormalities were subjected to digital radiographic imaging (100-cm focus-film distance; 56 kV; 200 mA; 5.0 mAs; 20 ms) in four projections (anteroposterior; right lateral in the neural position; right lateral in extension; and right lateral in flexion). To achieve the flexion and extension position, C7 was fixed rigidly, and a 55-Nm load was applied to C1. The parameters of intervertebral angle (IVA), lordosis angle (LA), anterior intervertebral height (aIVH), middle intervertebral height (mIVH) and posterior intervertebral height (pIVH) (Figure 1) were measured in each motion segment on every X-ray film using electronic vernier calipers (precision, 0.01 mm) and a protractor (precision, 0.1°). The data for each measurement were adjusted using a correction factor equal to (focus-film distance minus focus-object distance)/(focus-film distance).

**Figure 2:**
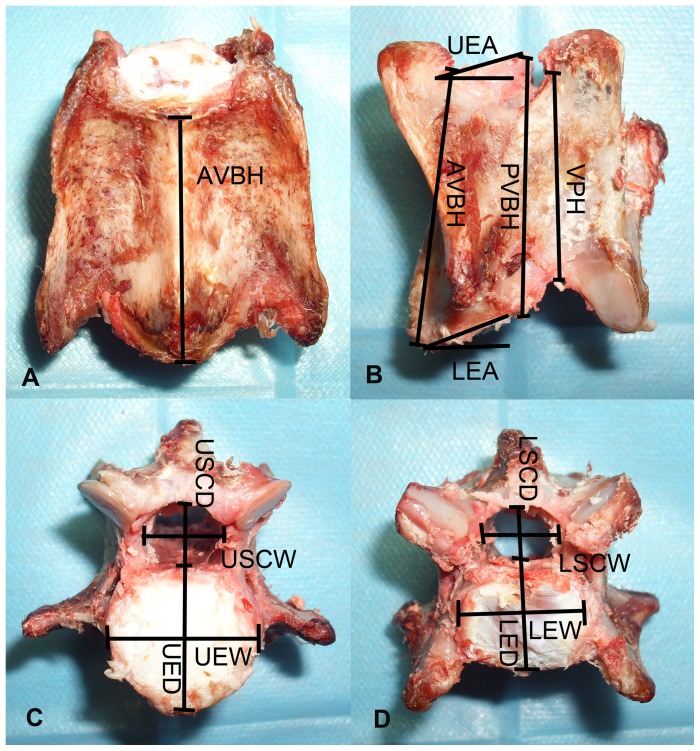
Anatomical measurements of goat cervical spines. A, Anterior vertebral body height (AVBH); B, AVBH, posterior vertebral body height (PVBH), vertebral pedicle height (VPH), upper and lower endplate angle (UEA and LEA); C, upper spinal cord depth and width (USCD and USCW), upper endplate depth and width (UED and UEW); D, lower spinal cord depth and width (LSCD and LSCW), lower endplate depth and width (LED and LEW).

After the radiological measurement, the anatomic evaluation was performed. Eleven linear and two angular parameters were measured using electronic vernier calipers and a protractor. The measurements included anterior vertebral body height (AVBH), posterior vertebral body height (PVBH), vertebral pedicle height (VPH), upper endplate width (UEW) and depth (UED), upper spinal canal width (USCW) and depth (USCD), lower endplate width (LEW) and depth (LED), lower spinal canal width (LSCW) and depth (LSCD), upper endplate angle (UEA) and lower endplate angle (LEA) (Figure 2).

### Radiological measurements of human cervical spines

Radiological measurements were performed on 50 normal human cervical spines (age, 50.5±8.6 years; range, 29–62 years; 28 women and 22 men) after the subjects provided written informed consent. The medical history of each human was reviewed to exclude any cervical skeletal disease, including trauma and metabolic disease, which may present anatomical and functional abnormalities. Digital X-ray films (100-cm focus-film distance; 56 kV; 200 mA; 5.0 mAs; 20 ms) were collected in four projections. To obtain the extension and flexion projection, the volunteers were asked to maintain the most extreme extension and flexion position that they could. The parameters of IVA, AL, aIVH, mIVH and pIVH were measured using electronic vernier calipers and a protractor. All of the data were multiplied by a correction factor.

### Design of the Artificial Cervical Vertebra and intervertebral Complex (ACVC)

**Figure 3:**
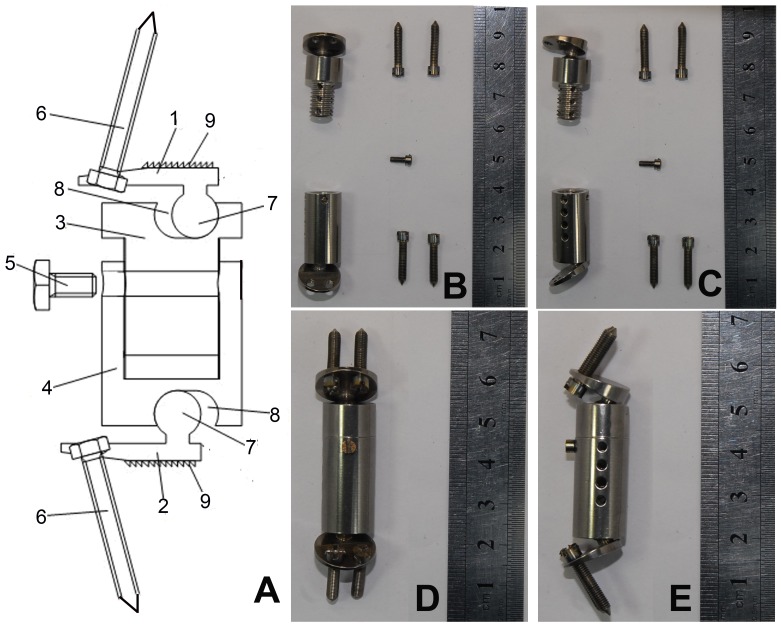
Scheme diagram and photos of the ACVC. A, Scheme diagram of the ACVC; B, actual photo of frontal view of the ACVC components; C, actual photo of lateral view of the ACVC components; D, actual photo of frontal view of the ACVC integer; F, actual photo of lateral view of the ACVC integer.

**Figure 4:**
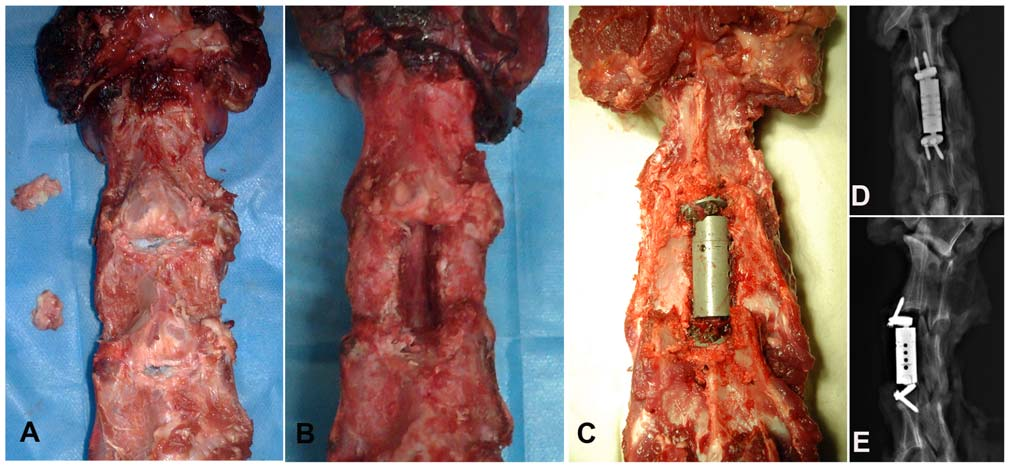
In vitro ACVC implantation in goat cervical spines. A, C2/3 and C3/4 discectomies; B, C3 subtotal corpectomy; C, fixation of ACVC; D and E, anterior-posterior and lateral X-ray films after the ACVC implantation.

**Figure 5:**
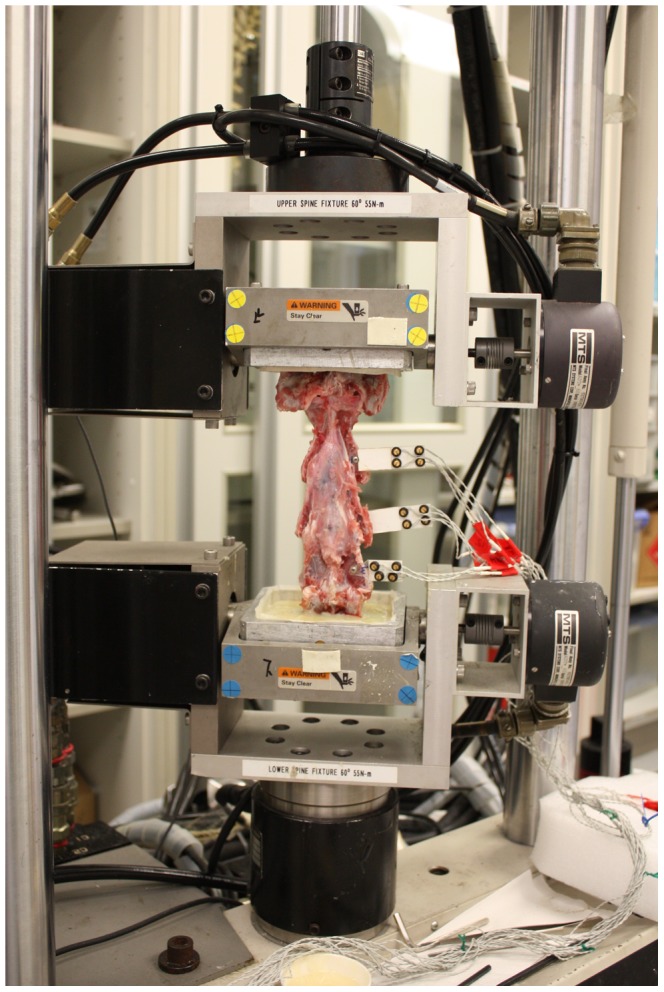
Biomechanical testing of ACVC implanted C1–C5 segments using a MTS machine.

**Figure 6:**
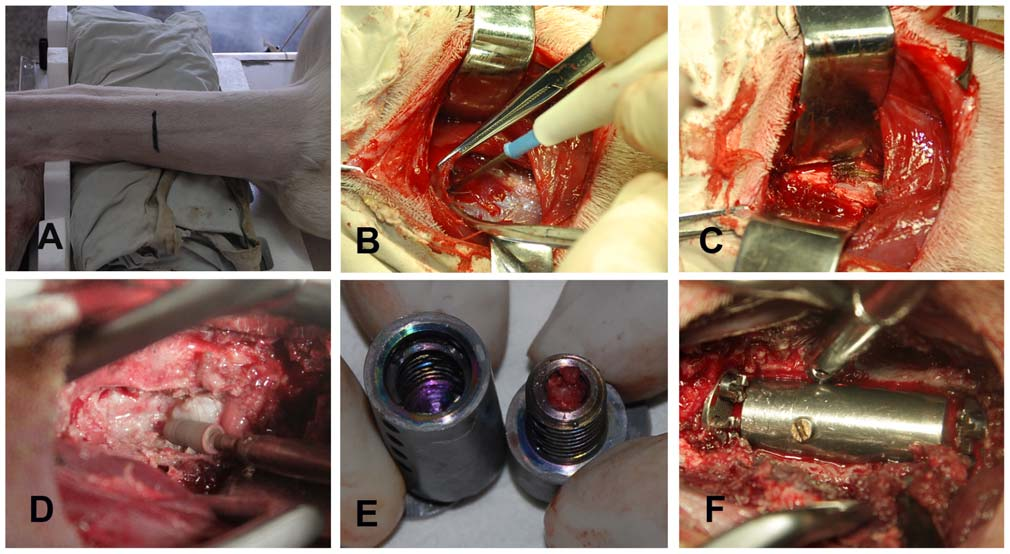
Surgical procedures for ACVC implantation through anterior approach in goat. A, The goat in a neutral supine position and the transverse incision at the C3 level; B, cutting the longus colli muscle; C, exposing the anterior surface of C2-4; D, C3 subtotal corpectomy using high-speed burr; E, filling the autologous bone into the hollow structure; F, affixing the ACVC with four screws.

The ACVC (China patent number: 201120225997.2; Figure 3) was composed of a titanium alloy (Ti6Al4V; Northwest Nonferrous Metal Research Institute, China) and consisted of an upper endplate component (Figure 3A-1), a lower endplate component (Figure 3A-2), an upper vertebral component (Figure 3A-3), a lower vertebral component (Figure 3A-4), one length-locking screw (Figure 3A-5), and four self-drilling trapping screws (Figure 3A-6). The dimensions of this artificial joint were based on the anatomical and radiological data from humans and goats, as appropriate.

**Figure 7:**
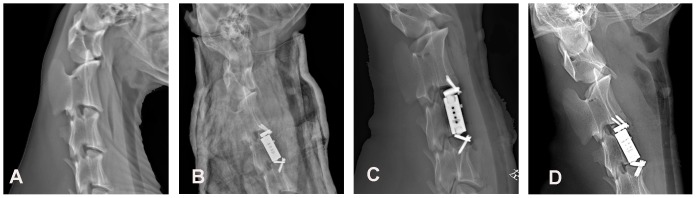
X-ray films before and after the in vivo ACVC implantation. A, Lateral X-ray film before the ACVC implantation; B, lateral X-ray film 3 weeks after the ACVC implantation; C, lateral X-ray film 6 weeks after the ACVC implantation; D, lateral X-ray film 12 weeks after the ACVC implantation.

**Figure 8:**
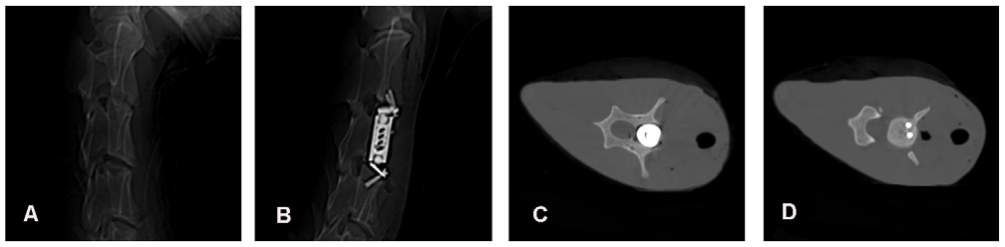
CT images of a goat in the ACVC group in vivo. A, CT image before the ACVC implantation shows the normal cervical spine; B, CT image 6 weeks after the ACVC implantation shows the location of ACVC in goat cervical spine; C, transverse image at the level of C3 6 weeks after the ACVC implantation shows the bony C3 vertebra hold the vertebral body component of the ACVC; D, transverse image at the level of C4 6 weeks after the ACVC implantation shows the screws went through the vertebral body of C4.

The ball-in-trough structure (Figure 3A-7 and 3A-8) is the most important part of this unconstrained metal-on-metal ACVC joint. In theory, the trough allows a 20° range of motion in flexion-extension, a 12° range of motion in lateral bending, a 360° range of motion in rotation, and a 1.5-mm anterior-posterior slide horizontally. The posterior column of the spine remains intact in this surgery, so the posterior column and surrounding soft tissues (ligaments and muscles) could limit all ranges of motion, especially rotation. Furthermore, the more-than-half ball-in-trough structure makes this joint more stable, without the possibility of dislocation.

The length of the vertebral components (Figures 3A-3 and 3A-4) can be changed and be locked during the surgery by using a length-locking screw (Figure 3A-5). This ensures that the ACVC can fit almost all patients. The vertebral components are hollow-structured, which allows the surgeon to implant the autologous bone in the hollow space and allows the surrounding bone to grow into the vertebral components through the holes to provide long-term stability.

**Figure 9:**
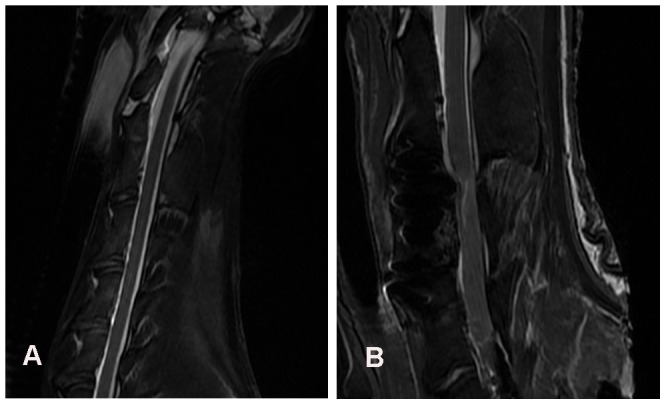
T2 weighted sagittal MR images of a goat in the ACVC group in vivo. A, MR image before the ACVC implantation shows the normal goat cervical spine ; B, MR image shows an artifact from the C2 to C4 vertebrae after the ACVC implantation.

The ACVC is fixed using four self-drilling trapping screws (Figure 3A-6). The screws are fixed toward the adjacent vertebral bodies at an angle of approximately 25°. There are many tiny teeth (Figure 3A-9) on the upper surface of the upper endplate component (Figure 3A-1) and the lower surface of the lower endplate component (Figure 3A-2), which improves the bond between the bony endplate and metallic endplate components.

### In vitro ACVC implantation in goat cervical spines and biomechanical testing

The cervical spines (C1–C5) were removed from 16 fresh adult goats (age, 24±4 months; average body weight, 52.6±3.6 kg; an equal number of females and males). The soft tissue was trimmed from the samples, leaving the ligaments intact. The samples were randomly divided into 2 groups: Group A, the control group (intact C2 to C4 segment, n = 8), and Group B, the ACVC group (which underwent anterior cervical discectomy, subtotal corpectomy, and ACVC implantation; n = 8). The purpose of this in vitro biomechanical experiment was to provide preliminary data for further establishing a goat model for ACVC implantation.

Based on the anatomical and radiological data, we chose C3 vertebrae and C2/3 and C3/4 intervertebral discs for the experiment. In Group B, C2/3 and C3/4 discectomies, C3 subtotal corpectomy, and ACVC implantation were performed.

The C2/3 and C3/4 intervertebral discs were cut transversely using a #11-blade scalpel and removed using nucleus pulposus forceps and curettes. The adjacent endplates were prepared by decorticating but without penetrating the subchondral surface. A 10-mm-wide trough was then drilled using a high-speed burr. The length from the inferior endplate of C2 to the superior endplate of C4 was measured using a gauge caliper. The length of the ACVC was adjusted, and the ACVC was placed in the trough in a neutral position. The trough was about 2-mm narrower than the vertebral components of the ACVC to ensure that it held the vertebral components of the ACVC tightly. The two superior and two inferior self-drilled trapping screws were fixed superoposteriorly and inferoposteriorly, respectively. X-ray films were taken to confirm the ACVC position (Figure 4). All of the samples were wrapped in saline-moistened towels and stored in plastic bags at −20°C until testing.

The biomechanical test was based on the principles that H.-J. Wilke reported [10]. Before testing, the frozen cervical specimens were thawed at room temperature. The end vertebrae of the specimens (C1 and C2, C4 and C5) were transfixed with perpendicular pins to enhance the fixation with mounting jigs, and the C1 to C5 vertebrae were then mounted in fast-drying epoxy resin (Huntsman Advanced Materials (HK) Limited, HK). The spinal construct was then inserted into a specially designed spinal fixture (MTS System Inc., Minneapolis, MN, USA). Each specimen was centered in the fixture with the center of axial rotation positioned just anterior to the spinal cord.

All of the data were recorded using a servohydraulic materials testing machine (MTS 858 Bionix machine, MTS System Inc., Minneapolis, MN, USA; Figure 5). During the testing, 0.9% saline was intermittently sprayed on the specimens to keep them moist.

During the in vitro kinematics tests, three infrared light-emitting diodes (LEDs) were rigidly attached to each vertebra from C2 to C4, serving as the definable points for three-dimensional motion (Figure 5). A marker carrier with 4 noncollinear light-emitting diodes on the base of the spine machine defined a general anatomical specimen coordinate system. Using this system, an anatomical coordinate system was defined, with the base of the spinal fixture set as the origin. An optoelectronic camera system (Optotrak 3020; Northern Digital, Waterloo, Canada) was used to measure the position markers at a sampling frequency of 20 Hz. Stereophotographs and 3D laser-scanning measurements of the markers fixed to the specimens were taken only during the fifth load cycle.

Three-dimensional flexibility tests were conducted on each of the specimens according to the protocol established by Zhu et al [11]. Spine testing was performed in a non-destructive manner. A multidirectional flexibility test was performed to determine the stability of the construct. A pure moment of 2.5-Nm was applied to the top vertebra (C1) while the specimen was allowed to move in an unconstrained 3-D manner. This continuous moment was applied at a rate of approximately 0.5°/second in all 3 primary directions of loading, namely flexion-extension, lateral bending, and axial rotation. The load was applied for 5 complete loading cycles. The first 4 cycles were used to precondition the specimen and minimize viscoelastic effects, and the fifth cycle was used for data analysis. The total angular range of motion was calculated for the last cycle.

The fatigue test consisted of 5,000 repetitions of axial rotation (fatigue load, 1.0 Nm; frequency, 0.25 Hz).

The kinematic behavior of each specimen was compared by examining the range of motion (ROM) and neural zone (NZ) of the C2–C4 segment of intact and ACVC-implanted goat spines from the fifth loading cycle before and after the fatigue test. The ROM in flexion-extension, lateral bending, and axial rotation was defined as rotation from the neutral position to a maximum load position. The NZ of the vertebral segment was calculated about the primary axis of rotation and described as the degree of rotation between the neutral position and the initiation point of spinal resistance to physiological motion.

### Establishment of a goat model of ACVC implantation

Sixteen adult goats (age, 22±4 months; average body weight, 53.8±4.9 kg; equal number of female and male goats) were employed to establish the goat model of ACVC implantation. The reasons to choose goats are their physiological and biomechanical similarity to those of human. The goats have a proper body size as well as cervical spine size for prosthesis implantation. Some studies have reported that goats are suitable animal model for cervical spine surgeries [12], [13], [14]. The 16 goats were randomly divided into two groups. Group A, the control group, consisted of 8 goats (age, 22±5 months; 4 males and 4 females); only the anterior vertebral surfaces were fully exposed, and the vertebrae, intervertebral disc and ligaments were not disturbed. Group B, the ACVC group, consisted of 8 goats (age, 22±4 months; 4 males and 4 females); in this group, ACVCs were implanted to establish the goat model of ACVC implantation.

### Detailed descriptions of the goat model establishment process

#### I. Animal preparations

X-ray films were obtained one week before the surgery to exclude cervical spine abnormalities using a C-arm fluoroscopic imager. Before surgery, all of the goats were kept without food and water for 24 hours to reduce the risk of aspiration and asphyxia during the surgery.

#### II. Anesthesia

All goats underwent general anesthesia as previously described [15]. Each animal was given 0.04 mg/kg of atropine subcutaneously, followed by 10 mg/kg of ketamine and 0.3 mg/kg of xylazine intramuscularly. After 10 minutes, 6 mg/kg of thiamylal sodium 2.5% solution was administered intravenously, and endotracheal intubation was carried out using an 8-mm-diameter tube. General anesthesia was maintained with approximately 1% halothane in O_2_.

#### III. Antibiotic

Cefazolin (50 mg/kg) was given intravenously (antibiotic prophylaxis) 30 minutes before the incision.

#### IV. Surgical procedures for anterior ACVC implantation in goats

The goats were placed in a neutral supine position. The anterolateral approach to the cervical spine was used. After the cervical area was shaved and the anterior neck was sterilely prepared, a transverse incision was made at the C3 level. The transverse incision line extended from the midline to a point 3 cm lateral on the right side (Figure 6A). The exposure was then widened and deepened. Generous subplatysmal dissection was performed for the ease of vertebral body exposure, and a sharp dissection was made between the fascial planes. The carotid sheath was identified by palpation and swept laterally, and the trachea and esophagus were retracted medially using a retractor. The longus colli muscle, which is well developed in goats, was then cut along the midline (Figure 6B). The C3 vertebral body was identified by palpation along the midline, and the disc space was identified (Figure 6C). The C2/3 level was verified by lateral x-ray films using a needle placed into the C2/3 intervertebral disc. At this point, all of the goats in control group were sutured.

The goat model was established in ACVC group, which underwent anterior C2/3 and C3/4 discectomy, C3 subtotal corpectomy, and ACVC implantation. Distracter pins were placed in the C2 and C4 vertebral bodies to open the disc space. The discectomies were initiated by making a transverse incision in the disc using a #11-blade scalpel. The C2/3 and C3/4 intervertebral discs were removed in piecemeal fashion using nucleus pulposus forceps and curettes. The cartilage on the inferior surface of the C2 endplate and the superior surface of the C4 endplate was removed, and the bony endplates were retained to provide bony fixation for four self-drilling trapping screws (Figure 3A-6) and to prevent graft subsidence. The depth of the spinal cord canal was defined by performing the discectomies above and below the intended corpectomy vertebrae, thereby making the corpectomy safer. A 10-mm-wide trough, which was narrower than the vertebral components of the ACVC (Figure 3A-3 and 3A-4) to ensure that it held the vertebral components of the ACVC tightly, was drilled using a matchstick-type high-speed burr under intermittent irrigation (Figure 6D). Careful movements were needed to ensure the complete decompression of the spinal cord without dural violation. Some bone from the C3 vertebral body was kept to fill the hollow structure in the vertebral body component of the ACVC (Figure 6E).

The length from the C2 inferior endplate to the C4 superior endplate was measured. The ACVC was then adjusted to that length and fixed using a length-locking screw (Figure 3A-5). The vertebral component of the ACVC was lightly and carefully inserted into the 10-mm-wide trough in a neutral position. The four self-drilling trapping screws were affixed to the C2 and C4 vertebral bodies in the following order: upper left, lower right, upper right, and lower left (Figure 6F). The distracter pins were removed, and a small amount of bone wax was placed into the distracter pinholes. A final x-ray was taken to verify the placement of the ACVC before closure.

The incisions were closed in layers with sutures. The goats were placed in plaster neck casts for 4 weeks to restrict their neck movement and facilitate the ACVC fixation.

### Postoperative observations

#### I. Animal care

Goat care was performed by veterinary physicians and trained animal care staff. After surgery, the goats were observed until they had fully recovered from anesthesia. Food and water intake was limited during the first 24 hours after surgery to reduce the risk of intestinal tympanites. A normal diet was established 2 days after surgery. Cefazolin (25 mg/kg) was administered twice a day for two days after the surgery.

After 4 weeks, when wound healing was completed, the neck plaster casts were removed and the goats were returned to their normal living quarters and allowed to carry out normal activity without restriction.

#### II. Clinical evaluation

Eating habits, ambulatory activities, health status, and neurological functions were monitored daily for the first 4 weeks and twice weekly for Weeks 5 to 12.

#### III. Radiological evaluation

Anteroposterior and lateral radiographic films were taken one week before the surgery and every three weeks postoperatively while the animals were under general anesthesia.

Computed tomography (CT) analyses were performed one week before the surgery and at the 6th and 12th week postoperation to determine the position of the prosthesis and the extent of bony fusion. Thin-cut (1.0-mm) contiguous slices in acquisition were obtained using a GE High Speed CT scanner (GE Medical Systems, Milwaukee, Wisconsin) in helical mode.

Magnetic resonance (MR) images of the goat cervical spines were obtained using a 1.5T system (Signa; GE Medical Systems, Milwaukee, Wisconsin) one week before the implantation and 6 weeks after the implantation, with the animal under general anesthesia. MR images of the same resolution were also obtained for the cervical spines isolated en bloc immediately after sacrifice. T1W fast spin echo sagittal images were acquired with the following imaging parameters: TR/TE = 400/20 msec, matrix = 256×256, section thickness/interslice gap = 4.5/0.5 mm, echo train length (ETL, turbo factor) = 2, and field of view = 24 cm. T2W fast spin echo saggital images were acquired with the following imaging parameters: TR/TE = 3000/80 msec, matrix = 256×256, section thickness/interslice gap = 4.5/0.5 mm, echo train length (ETL, turbo factor) = 16, and field of view = 24 cm.

The radiographs were evaluated by two evaluators for fusion, graft extrusion, and bone fracture or collapse.

#### IV. Euthanasia

All of the animals were observed for 12 weeks before being killed with an overdose of pentobarbital (200 mg/kg).

### Biomechanical evaluation

Immediately after sacrifice, the C1–C5 motion segments were dissected from the harvested cervical spines and cleaned of residual soft tissue, with care taken not to disturb the spinal bony and ligamentous attachments. The specimens were wrapped in gauze soaked in 0.9% saline and kept frozen at −20°C in polyethylene bags until testing. The biomechanical evaluation was performed as described above.

### Statistical analysis

All results were analyzed using SPSS Version 13.0 (Chicago, IL). The results are presented as the means ± SDs. The data for ROM and NZ were analyzed using the two-tailed Student's t-test. P values less than 0.05 were considered statistically significant.

## Results

### Radiological data of goat and human cervical spines (Tables 1, 2, 3, 4, and 5)

The intervertebral angles (IVAs) of the human cervical spines were not less than those of the goat cervical spines in the neutral, flexion, and extension positions (Tables 1, 2, and 3). The IVAs in the neutral position at the C2–C5 levels in goats and humans were not significantly different (Table 1). Although the human IVAs were greater than those of the goats at C3–C4 in the flexion position and at C2–C4 in the extension position, the range of IVA motions (IVA in extension minus IVA in flexion) in goats and humans was not significantly different at C2–C6 (Table 4).

**Table 1:** Intervertebral angle (IVA) and lordosis angle (LA) for goat and human cervical spines in the neutral position.

	IVA in neutral position (°)		LA in neutral position (°)	
	Humans	Goats	Humans	Goats
C2–C3	6.1±4.9	5.3±2.3	3.1±2.2	0.8±2.4*
C3–C4	9.6±4.7	8.6±3.1	5.3±4.9	4.1±3.1
C4–C5	10.1±2.9	9.2±3.0	7.2±2.4	7.1±2.6
C5–C6	10.9±2.1	8.9±3.9*	8.1±3.9	11.3±3.9*
C6–C7	9.1±2.3	8.1±2.2*	8.2±3.7	19.8±4.0*

+ = lordosis, − = kyphosis.

* P<0.05 versus the human spines.

**Table 2:** Intervertebral angle (IVA) and lordosis angle (LA) for goat and human cervical spines in flexion.

	IVA in flexion (°)		LA in flexion (°)	
	Humans	Goats	humans	Goats
C2–C3	4.3±3.9	3.2±2.9	−2.1±3.4	−0.8±3.2
C3–C4	4.2±2.9	3.0±2.6*	−2.8±2.1	−0.6±3.3*
C4–C5	2.8±2.1	2.2±1.9	−3.6±3.0	−0.6±3.7*
C5–C6	3.1±2.0	2.4±2.1	−3.5±3.6	0.3±5.1*
C6–C7	3.5±3.1	3.4±3.2	−3.1±2.6	3.3±4.9*

+ = lordosis, − = kyphosis.

* P<0.05 versus the human spines.

**Table 3:** Intervertebral angle (IVA) and lordosis angle (LA) for goat and human cervical spines in extension.

	IVA in extension (°)		LA in extension (°)	
	Humans	Goats	Humans	Goats
C2–C3	8.1±4.2	6.5±3.4*	8.3±4.8	2.2±3.3*
C3–C4	11.3±4.9	9.6±3.1*	13.4±4.9	6.1±3.6*
C4–C5	13.2±2.4	12.1±4.6	12.3±3.7	11.3±4.0
C5–C6	12.1±3.9	11.3±3.9	15.2±4.3	15.1±3.9
C6–C7	12.2±3.7	11.8±4.0	14.3±4.1	23.1±4.7*

+ = lordosis, − = kyphosis.

* P<0.05 versus the human spines.

**Table 4:** Total motion of the intervertebral angle (IVA) and lordosis angle (LA) (extension minus flexion) for goat and human cervical spines.

	Total motion of IVA (°)		Total motion of LA (°)	
	Humans	Goats	Humans	Goats
C2–C3	3.8±1.8	3.3±1.2	10.4±2.1*	3.0±2.4
C3–C4	7.1±1.7	6.6±1.1	16.2±2.6*	6.7±3.1
C4–C5	10.4±1.9	9.9±1.5	15.9±3.4*	11.9±3.5
C5–C6	9.0±1.2	8.9±1.3	18.7±3.1*	14.8±2.8
C6–C7	8.7±1.3*	8.4±1.2	17.4±2.9*	19.8±3.9

+ = lordosis, − = kyphosis.

* P<0.05 versus the human spines.

The lordosis angles (LAs) of C2–C6 in humans were not less than that of C2–C6 in goats, while the LA of C6–C7 in humans was less than that of C6–C7 in goats in the three positions (Tables 1, 2, and 3). There was no significant difference between the LA in goats and humans in the neutral position at the levels of C3-4 and C4-5 (Table 1). The total motion of the LA (LA in extension minus LA in flexion) for C2–C7 was significantly higher in humans than in goats (Table 4).

The endplates of human vertebrae are all concave. The middle intervertebral height (mIVH) of all of the cervical spines was greater than both the anterior and posterior intervertebral heights (aIVH and pIVH) of the cervical spines (Table 5). In goats, however, the intervertebral height decreases from the front to the back because the superior cervical endplates are convex and the inferior endplates are concave. The mean IVHs of goat cervical spines are significantly greater than those of human cervical spines.

**Table 5:** Intervertebral height (IVH) of goat and human cervical spines.

	aIVH (mm)		mIVH (mm)		pIVH (mm)		Mean IVH (mm)	
	Humans	Goats	Humans	Goats	Humans	Goats	Humans	Goats
C2/3	5.1±0.6	6.6±0.9*	5.6±0.3	6.2±0.5*	3.8±0.4	4.9±0.8*	4.8±0.5	5.9±0.7*
C3/4	5.5±0.2	6.8±0.5*	6.2±0.4	6.1±0.4	3.9±1.2	4.5±1.0*	5.2±0.4	5.8±0.6*
C4/5	5.3±0.5	7.2±1.1*	6.0±0.6	5.7±0.3*	3.5±0.6	3.9±0.5*	4.9±0.3	5.6±0.4*
C5/6	4.7±0.4	7.5±0.6*	5.9±0.9	5.6±0.4*	3.5±0.3	3.8±0.7*	4.7±0.6	5.6±0.8*
C6/7	5.1±1.2	7.9±0.3*	5.8±0.5	5.0±0.9*	3.8±0.7	4.1±1.1	4.9±0.8	5.7±0.6*

* P<0.05 versus the human spines.

### Anatomical data for goat cervical spines (Table 6)

**Table 6:** Anatomical data of goat cervical spines (mean ± SD).

	C2	C3	C4	C5	C6	C7
AVBH (mm)	46.0±2.5	38.2±1.4	36.7±3.0	34.9±4.0	29.6±1.9	20.6±1.3
PVBH (mm)	44.2±1.8	37.3±1.8	34.9±2.2	32.6±1.2	27.4±1.6	19.3±2.1
VPH (mm)	31.5±2.3	25.1±2.1	22.1±1.6	20.7±3.8	16.1±2.3	13.9±1.4
UED (mm)	N/A	18.4±1.3	18.8±0.9	18.5±1.2	18.4±1.0	17.6±1.3
UEW (mm)	N/A	20.2±3.2	20.7±1.5	20.4±1.7	17.0±1.3	15.3±2.1
USCD (mm)	14.4±1.0	11.6±0.5	11.3±0.4	11.9±0.8	11.4±2.0	10.9±1.9
USCW (mm)	15.2±1.1	14.0±1.2	14.1±0.8	15.2±2.1	16.3±0.7	15.3±2.2
LED (mm)	20.6±1.6	21.4±1.4	22.2±2.7	22.1±1.0	20.6±1.2	21.0±2.5
LEW (mm)	22.9±2.7	24.3±1.8	24.0±1.0	24.6±1.4	22.0±1.4	21.1±2.3
LSCD (mm)	11.7±0.5	11.6±0.9	11.9±0.7	12.5±0.7	13.6±1.6	13.2±1.0
LSCW (mm)	13.2±0.9	11.9±0.3	12.1±0.6	13.9±1.9	16.0±2.0	16.3±1.9
UEA (°)	N/A	52.6.0±4.9	54.6±7.3	55.5±6.5	57.5±4.0	58.3±5.1
LEA (°)	66.5±4.4	65.0±4.6	64.5±4.9	60.9±6.6	59.5±3.4	58.7±4.9

The vertebral body height (VBH) and vertebral pedicle height (VPH) of the goats decreased from C2 to C7. The width of both the superior and inferior endplates was larger than the depth of the endplates. The areas of the endplates were consistent from C2 to C7. The width of the spinal canal, both superiorly and inferiorly, was larger than the depth. The upper endplate angle increased from C2 to C7, whereas the lower endplate angle decreased from C2 to C7.

### Design of the artificial cervical vertebrae and intervertebral complex (ACVC)

Figures 3B, C, D and E are actual photos of frontal and lateral views of the ACVC components and integer. Based on the anatomical data for the goat cervical spines, the entire length of the ACVC, except for the self-drilling trapping screws, was 46 mm, which can be adjusted to a maximum of 52 mm. The four self-drilling trapping screws are at an angle of approximately 75° with the endplate components superoposteriorly and inferoposteriorly. The anterior-posterior slide design allows the artificial joint to more closely approximate the physiological movement and biomechanics of the normal cervical spine, which may decrease the possibility of postsurgery degeneration.

### In vitro ACVC implantation

The C2/3 and C3/4 intervertebral discs and the cartilage on the inferior surface of C2 and the superior surface of C3 were removed (Figure 4A). A 10-mm-wide trough was made in the middle of the C3 vertebral body using a high-speed burr (Figure 4B). The length of the ACVC was adjusted, and the implant was impacted into the trough in the C3 vertebral body. Four screws were bored into the adjacent vertebral bodies in superoposterior or inferoposterior directions (Figure 4C). X-ray films were taken (Figure 4D and 4E) and indicated that the position and length of the ACVC were suitable and did not compress the spinal cord. The four self-drilling trapping pins were affixed to the adjacent vertebral bodies through the endplates. The goat cervical vertebrae were in normal alignment.

### In vivo anterior ACVC implantation surgery

#### I. Clinical evaluation

All of the goats successfully underwent surgery under general anesthesia, which was appropriate for the surgery. After the administration of the general anesthesia, the goats laid motionless on the operating table during the surgery. All of the surgeries on goats were successful and the goats appeared to tolerate ACVC very well during the experiment. One goat in experimental group died after undergoing anesthesia for radiological evaluation.

#### II. Radiological evaluation

X-ray films were taken 1 week before the surgery (Figure 7A), 3 weeks after the ACVC placement (while the goats were in plaster neck casts, Figure 7B), 6 weeks after the ACVC placement (Figure 7C) and 12 weeks after the ACVC placement (Figure 7D) in the experimental group (the ACVC group). The skeletal structures of the goats were normal before the surgery. The location of the ACVC was good after the surgery. We identified that, in 4 out of 7 goats in the experimental group, there was a small gap between the ACVC and the C3 vertebral body 6 weeks after the surgery, which disappeared 12 weeks after the surgery. No bone fractures, joint dislocation, or screw loosening were found during the experimental period.

Figure 8 shows the CT images of a goat in the ACVC group preoperation (Figure 8A) and 6 weeks after surgery (Figure 8B) and transverse images at the level of C3 (Figure 8C) and C4 (Figure 8D) at 6 weeks. We clearly see the shape and location of the ACVC. The position of the vertebral body component of the ACVC was at the C3 vertebral body close to the spinal canal. The screws directed superior-posteriorly into the C2 vertebral body and inferior-posteriorly into the C4 vertebral body (Figure 8B) were visible. The vertebral body component of the ACVC was located in the anterior and middle column of the spine. The residual bony C3 vertebral body held the vertebral body component of the ACVC tightly (Figure 8C). The screws went through the vertebral bodies of C4 (Figure 8D) and C2 (not shown).

We used fast spin echo MR sequence to scan the goat cervical spines before and after ACVC implantation. Fast spin echo sequence has been proven to decrease the artifact produced by metallic implants [16]. Before the surgery, we could clearly see the goat's spinal cord, vertebral body and intervertebral disc (Figure 9A). After the surgery, we still can see the spinal cord. But we can see an artifact from the C2 to C4 vertebrae in which the ACVC was implanted, and we could not see the detail structure of ACVC (Figure 9B).

### Kinematics results

#### I. In vitro ACVC implantation (Table 7)

**Table 7:** Ranges of motion (ROM) and neutral zone (NZ) profiles of the C2 to C4 segments of the control group and the ACVC group before and after the fatigue test in all directions in in vitro ACVC implantation.

Motion	Control group (n = 8)		ACVC group (n = 8)	
	Before fatigue test	After fatigue test	Before fatigue test	After fatigue test
Range of Motion (in Degrees)				
Flexion-extension	6.2±0.9	7.1±0.8	8.7±1.2^a^	10.0±1.3^b^
Lateral bending	10.2±0.8	11.2±1.1	17.4±1.8^a^	19.5±2.2^b^
Rotation	12.1±1.1	11.0±1.3	23.5±2.2^a^	25.4±2.1^b^
Neural Zone (in Degrees)				
Flexion-extension	1.0±0.4	1.4±0.8	1.7±0.7^a^	2.2±0.6^b^
Lateral bending	0.9±0.3	1.2±0.3	3.2±1.8^a^	5.2±2.0^b^
Rotation	1.2±0.3	1.1±0.3	5.2±1.2^a^	6.6±1.7^b^

a P<0.05 versus the control group before fatigue test.

b P<0.05 versus the control group after fatigue test.

The ROMs of C2 to C4 in the control group (no ACVC implantation in vitro) in response to 2.5-Nm before the fatigue test were 6.2±0.9° in flexion-extension, 10.2±0.8° in lateral bending, and 12.1±1.1° in rotation. The ROMs of C2 to C4 in the ACVC group (ACVC implantation in vitro) in response to a 2.5-Nm load before the fatigue test were 8.7±1.2° in flexion-extension, 17.4±1.8° in lateral bending, and 23.5±2.2° in rotation. The ROMs of the ACVC group were larger than those of the control group. The ROM in rotation was the most increased (by 1.94-fold; 23.5/12.1), whereas the ROM in flexion-extension was the least increased (by 1.40-fold; 8.7/6.2). There was no significant difference between the ROM before and after the fatigue test.

The NZs of C2 to C4 in the control group in response to 2.5-Nm before the fatigue test were 1.0±0.4° in flexion-extension, 0.9±0.3° in lateral bending, and 1.2±0.3° in rotation. The NZs of C2 to C4 in the ACVC group in response to 2.5-Nm before the fatigue test were 1.7±0.7° in flexion-extension, 3.2±1.8° in lateral bending, and 5.2±1.2° in rotation. The NZs of the ACVC group were greater than those of the control group. The NZ in rotation was the most increased (by 4.33-fold; 5.2/1.2), whereas the NZ in flexion-extension was the least increased (by 1.70-fold; 1.7/1.0). No significant difference was found in the NZ before and after the fatigue test.

#### II. In vivo ACVC implantation (data shown in Table 8)

**Table 8:** Ranges of motion (ROM) and neutral zone (NZ) profiles of the C2 to C4 segments of the control group and the ACVC group before and after the fatigue test in all directions in in vivo ACVC implantation.

Motion	Control group (n = 8)		ACVC group (n = 7)	
	Before fatigue test	After fatigue test	Before fatigue test	After fatigue test
Range of Motion (in Degrees)				
Flexion-extension	6.5±0.9	6.9±0.8	6.2±0.7	6.0±1.3
Lateral bending	9.6±1.1	10.6±1.5	9.1±1.5	10.3±1.3
Rotation	11.2±0.8	11.6±1.1	17.5±1.3^a^	18.2±1.8^b^
Neural Zone (in Degrees)				
Flexion-extension	1.0±0.6	1.1±0.7	1.5±0.7	2.3±1.1
Lateral bending	1.1±0.4	1.7±0.7	2.1±1.3	2.8±1.2
Rotation	1.0±0.3	1.2±0.3	3.4±0.7^a^	4.5±1.5^b^

a P<0.05 versus the control group before fatigue test.

b P<0.05 versus the control group after fatigue test.

The ROMs of C2 to C4 in the control group (no ACVC implantation in vivo) in response to a 2.5-Nm load before the fatigue test were 6.5±0.9° in flexion-extension, 9.6±1.1° in lateral bending, and 11.2±0.8° in rotation. The ROMs of C2 to C4 in the ACVC group (ACVC implantation in vivo) in response to 2.5-Nm before the fatigue test were 6.2±0.7° in flexion-extension, 9.1±1.5° in lateral bending, and 17.5±1.3° in rotation. Before the fatigue test, no significant difference was found for the ROM in flexion-extension between the control and ACVC groups, nor was a significant difference found for the ROM in the direction of lateral bending between the control and ACVC groups. The ROM in rotation of the ACVC group was larger than that of the control group. There was no significant difference in the ROM values before and after the fatigue test. The results after the fatigue test were similar to those found before the fatigue test.

The NZs of C2 to C4 in the control group in response to 2.5-Nm before the fatigue test were 1.0±0.6° in flexion-extension, 1.1±0.4° in lateral bending, and 1.0±0.3° in rotation. The NZs of C2 to C4 in the ACVC group in response to 2.5-Nm before the fatigue test were 1.5±0.7° in flexion-extension, 2.1±1.3° in lateral bending, and 3.4±0.7° in rotation. No significant difference was found in NZ in the direction of flexion-extension between the control and ACVC group before the fatigue test or in NZ in the direction of lateral bending. The NZ in rotation was greater in the ACVC group than in the control group. Similar results were found after the fatigue test. No significant difference was found in the NZ values before and after the fatigue test.

## Discussion

### Comparison of radiological and anatomical data of cervical spines between goats and humans

The purpose of the measurements was to collect anatomical data relevant to the design of artificial joints for humans and goats and to determine the optimal spinal segment for which the characteristics are similar in human and goats.

As reported in the Results section, the radiological data of the IVA and LA in the neutral position show similarities for the IVA in goats and humans at the level of C2-3, C3-4, and C4-5 and for the LA at the level of C3-4 and C4-5. The functional radiological data demonstrate the similarity of the IVA in total motion between goats and humans from C2 to C6 and significant differences in the LA in total motion between goats and humans at all cervical levels. Because of the geometric difference between goat and human endplates, all mean IVHs of the goat cervical spine are greater than those of the human cervical spine. The average IVHs of the goat cervical spine range from 0.6 to 1.1 mm, which is 12% to 23% greater than the average IVHs in humans.

By comparing the human cervical spine anatomical data reported in other studies [17], [18], [19], [20] with our goat cervical spine anatomical data, we found that there are some anatomical differences between human and goat cervical spines. Goats have taller, conical cervical vertebral bodies, convex upper endplates, and concave lower endplates, while humans have wider, more cylindrical vertebral bodies and concave upper and lower endplates. According to the anatomical data on human and sheep cervical vertebrae reported by Kandziora [20], both the anterior and posterior vertebral body heights in goats are approximately 2 to 3 times larger than those of human C2 to C7 vertebrae, while the anterior and posterior vertebral body heights of C3 to C7 in goats are somewhat smaller than those of sheep. The upper and lower endplate widths and depths of C3 to C7 in goats are smaller than those of sheep, while those of goats are similar to those of humans. The upper and lower spinal canal widths and depths of C3 to C7 in goats are similar to those of sheep, and the spinal canal areas of C3 to C7 in goats are smaller than those of humans.

### Design of the ACVC

#### I.Materials

Several types of materials, including titanium, titanium alloys, cobalt-chromium, stainless steel, ceramics, and ultrahigh-molecular-weight polylene (UHMWPE), have been widely used in the manufacture of artificial joints. Each of these materials has advantages and disadvantages. The most important priorities for a material used to produce a permanent implant are biocompatibility and mechanical stability.

Biocompatibility is defined as the ability to function in vivo without eliciting intolerable local or systematic responses in the body. Metal alloys are used to balance the unwanted qualities of one metal with the desirable features of another. Titanium alloys have been widely used in implants. The Ti6Al4V alloy is the most widely used titanium alloy. The Ti6Al4V alloy has several good properties, including wear resistance, resistance to corrosion and degradation, adequate mechanical properties, valuable imaging characteristics, and a low elasticity modulus. A great concern in joint replacement is osteolysis, cytokine production, and the inflammatory response of wear-related particulate debris. Metal-on-metal peripheral joint arthroplasties have shown no to minimal osteolysis, produce less debris than UHMWPE, and elicit a lower inflammatory response, although metal-on-metal peripheral joint replacements have been associated with an elevated serum concentration of metal ions and hypersensitivity-related lymphocytic responses.

#### II.Articulation

The advantages of unconstrained devices include their allowance of normal or near-normal physiological motions and their potential to reduce the stress at the bone-implant interface. Although the unconstrained devices present greater opportunities for dislocation, our ACVC will not dislocate because its articulation is closed. The flexion-extension and lateral bending of the ACVC is controlled more by the prostheses itself and less by the surrounding soft tissue and posterior column structures of the spine. The ACVC has a lower ROM and greater stress in flexion-extension and lateral bending at the plate-bone interface, which is important for long-term stability. The rotation of the ACVC is mostly controlled by the posterior column structures of the spine and the surrounding soft tissue.

Unlike the ProDisc-C, the most constrained ball-in-socket articulation device among the current cervical artificial disc replacements, our ACVC has a ball-in-trough articulation. The ball-in-trough articulation allows anterior-posterior translation independent of flexion-extension, which ball-in-socket articulation can not provide. This is an important aspect in ACDR with respect to kinematics, such as range of motion (ROM). In most joints, normal movement involves a combination of sliding, flexion/extension, lateral bending and rotation.

The ACVC was designed to introduce few kinematic changes and have kinematics which are as close to those of the normal cervical spine as possible. Another matter of similar importance is eliminating disease components of the cervical spine, such as herniated discs or spondylotic sites, and minimizing bone sacrifice while providing sufficient primary and secondary implant-bone anchorage. The primary fixation of the ACVC is achieved via screws and a trough in the vertebral body that clamps the ACVC. The four screws are placed superior-posteriorly and inferior-posteriorly, creating the strongest possible fixation for the ACVC. For secondary fixation, bone ingrowth is encouraged by the endplate components of the ACVC, which provide a rough, grit-blasted surface, and by the holes in the vertebral body component of the ACVC.

#### III.Axis of rotation (AOR)

Another critical feature is the localization of the axis of rotation in artificial joints and whether it matches the physiological center of rotation of an implanted segment. The ball-in-trough articulation allows the articulation to slide forward and backward. Because the ACVC is an unconstrained joint, the AOR of the ACVC is variable within the intervertebral space. The ACVC is designed to locate the AOR in the posterior half of the vertebral body, which is similar to its location in the intact cervical spine.

### Animal model

For the biomechanical study of artificial joints in the spine, human cadavers [21], [22] and animal models have been well studied. Human cadavers have the same biomechanical properties as living humans, but they are precious and their use raises ethical issues; furthermore, they cannot be used to evaluate the in vivo reactions of human vertebrae with artificial joints.

Animal models are essential for investigating the biocompatibility, safety, in vivo tissue response and biomechanical functions of artificial joints prior to human trials. To date, no single animal model has sufficiently reproduced the human spine's biomechanical and physiological properties.

Several selection factors led us to choose goats as the appropriate animal model for testing the ACVC: their biological characteristics, which are analogous to those of humans (physiological properties of the cervical spine); bone tissue microarchitecture (bone microstructure and composition, as well as bone modeling and remodeling properties); tolerance to surgery; resistance to infection and disease; ease of pre- and postoperative maintenance; availability; and cost. Two requirements for an appropriate animal model are the ability to replicate the surgical technique and outcomes that are similar to those of humans [23], [24]. The assessment of the biomechanics associated with our implant was of interest in this study, so the larger, more expensive animals were more suitable because they more closely approximate the size and bony anatomy of humans. To date, the dog model has been one of the most commonly used animal models in biomechanical studies. However, some of the drawbacks of dog models are that dogs have a larger range of motion in the cervical spine compared with humans; that solid fusion occurs in almost all dogs, unlike in humans; and that dogs spend almost their entire lives with their heads in a flexed position. Compared with dogs, sheep and goats are considered good animal models for spine biomechanics analysis. Sheep and goats have a suitable body size for the implantation of implants and prostheses [12], [25]. AI Pearce [26] reviewed several animal models used in orthopedic research and found that sheep and goats are the most similar animals to humans in terms of macrostructure and that they are moderately similar to humans in terms of bone composition and bone remodeling. Furthermore, sheep and goats keep their heads upright throughout the majority of their lifetimes, which makes their cervical spine physiologically and biomechanically similar to that of humans. There is little information comparing the utility of goats versus sheep for implant-related studies. Therefore, the choice of which small ruminant to use most likely depends on availability and other factors. Compared with sheep, goats have a smaller body size, and, to some extent, goats are easy to manipulate pre- and post-surgery. Anderson ML [12] reported that the goat is a suitable animal model for testing human implants and materials, as they are considered to have a metabolic rate and bone remodeling rate similar to those of humans. Leung KS [13] reported that in certain regions characterized by high temperatures and humidity, such as south-east Asia, goats are reported to be more tolerant to ambient conditions than other species, such as sheep. Zdeblick TA [14] also stated that the goat is an excellent model for anterior cervical spine fusion procedures.

Furthermore, based on the radiological and anatomical data on goat cervical spines, we believe that the goat cervical spine, especially from C2 to C4, is appropriate to replicate the biomechanical properties in humans. The goat is an excellent animal model for studying the biomechanical properties of cervical spine implants in vivo.

All of the goats tolerated the surgeries very well, with the exception that one died from anesthesia complications. The general anesthesia provided a good condition for surgeries. Because of the structure of the gastrointestinal system of goats, it is very important for the animals to undergo fasting for 24 hours before the surgery and to limit the food uptake 24 hours after the surgery to avoid the risk of aspiration and asphyxia during the surgery and intestinal tympanites after the surgery. Several different surgical techniques using the anterior approach have been developed to relieve the symptoms of pain. The anterior approach to remove herniated discs is safe and efficient in decompressing both the spinal cord and cervical nerve roots. Therefore, we chose to implant the ACVC using the anterior approach.

The radiological results showed that the ACVC firmly stayed in position without extrusion. The ACVC successfully restored the intervertebral disc height, foraminal height, and cervical alignment (without causing kyphosis or lordosis deformity). There was no sign of fracture, subsidence, or loosening.

### Kinematics data

To preserve the motion in the degenerative segment of the cervical spine after the anterior cervical discectomy and subtotal corpectomy, we designed this ACVC implant and established the goat model for ACVC implantation via the anterior cervical approach.

Before the in vivo ACVC implantation, we performed the in vitro implantation and the fatigue test to ensure the longevity of the ACVC. We found that there was no significant difference in ROM and NZ before and after the fatigue test, and there was no loosening of the fixation screws. The in vivo implantation experiment demonstrated that the ACVC is safe in goats. Compared with the intact state (Table 9), in vitro implantation of the ACVC led to increased ROM in all three directions (flexion-extension, lateral bending, and rotation). In vivo implantation of the ACVC for 12 weeks resulted in no significant differences in ROM during flexion-extension and lateral bending and increased ROM during rotation, albeit to a lesser extent than in vitro ACVC implantation (1.56-fold in vivo versus 2.10-fold in vitro during rotation). Regarding the NZ, which represents the portions of the ROM in which there is ligamentous/hardware laxity, after in vitro implantation, the NZ increased in all directions, whereas only the NZ in rotation was increased after 12 weeks of in vitro implantation. The proposed reasons for these kinematics changes are as follows. The design of the ACVC in flexion-extension and lateral bending is constrained by both the articulation structure and the posterior column of the cervical spine, whereas the motion in rotation is only restricted by the posterior column. Twelve weeks after in vivo implantation, the ROM and NZ in flexion-extension and lateral bending were not different from those of the intact state. Furthermore, compared with the ACVC implantation in vitro, the ACVC in vivo implantation significantly decreased the ROM in all directions and NZ in rotation. This is because discectomy was performed more thoroughly in vitro than in vivo. Furthermore, there was more fibrous tissue after the surgery, which may have restricted the motions. When designing this ACVC, our primary aim was to facilitate C2-4 flexion and extension, which are, in theory, the most important functions for preserving the motion and preventing degeneration of the adjacent segments. Furthermore, C0–C2 undergo the greatest degree of axial rotation [27], [28]. Therefore, even though the ROMs and NZs were more greatly increased following in vitro implantation than in vivo implantation, we still believe the ACVC will prove beneficial in vivo by preserving the motion after discectomy and subtotal corpectomy.

**Table 9:** Ranges of motion (ROM) and neutral zone (NZ) profiles of the C2 to C4 segments of the control group in in vivo implantation, the ACVC in vitro implantation group and the ACVC in vivo implantation group before the fatigue test in all directions.

Motion	Control group (n = 8)	ACVC in vitro group (n = 8)	ACVC in vivo group (n = 7)
Range of Motion (in Degrees)			
Flexion-extension	6.5±0.9	8.7±1.2^a^	6.2±0.7^b^
Lateral bending	9.6±1.1	17.4±1.8^a^	9.1±1.5^b^
Rotation	11.2±0.8	23.5±2.2^a^	17.5±1.3^a^^,^ b
Neural Zone (in Degrees)			
Flexion-extension	1.0±0.6	1.7±0.7^a^	1.5±0.7
Lateral bending	1.1±0.4	3.2±1.8^a^	2.1±1.3
Rotation	1.0±0.3	5.2±1.2^a^	3.4±0.7^a^^,^ b

a P<0.05 versus the control group (in vivo implantation).

b P<0.05 versus the ACVC in vitro group.

The control group in in vitro implantation is similar to the control group in in vivo implantation. The data for the control group in in vitro implantation are not shown in the table below. The data from before and after the fatigue test were not significantly different, and the data for these groups after the fatigue test are not shown in the table below.

Considering that the cycles of fatigue test in the in vitro implantation experiment were limited and the degree of fatigue was considerably lower than that experienced under physiological conditions, we also performed the fatigue test in the in vivo implantation specimens. Additionally, there was no notable difference in ROM and NZ before and after the fatigue test and the loosening of the fixation screws. All of the above data indicate that the ACVC could provide good immediate stability.

In conclusion, based on radiological and anatomical data on goat and human cervical spines, we chose C2–C4 in goats as the segments most similar to those of humans. Therefore, those segments were the most optimal segments to establish an excellent goat model for anterior-approach ACVC implantation and test the biomechanical characteristics of ACVC. The clinical, radiological, and biomechanical evaluations confirmed that the ACVC provided immediate stability and preservation of motion.
